# Immunotherapy in melanoma: advances, pitfalls, and future perspectives

**DOI:** 10.3389/fmolb.2024.1403021

**Published:** 2024-06-28

**Authors:** Cristina Sorino, Simona Iezzi, Ludovica Ciuffreda, Italia Falcone

**Affiliations:** SAFU, Department of Research, Advanced Diagnostics, and Technological Innovation, IRCCS-Regina Elena National Cancer Institute, Rome, Italy

**Keywords:** melanoma, immunotherapy, immune checkpoint inhibitors (ICIs), adoptive cell therapy (ACT), precision medicine

## Abstract

Cutaneous melanoma is the deadliest and most aggressive form of skin cancer owing to its high capacity for metastasis. Over the past few decades, the management of this type of malignancy has undergone a significant revolution with the advent of both targeted therapies and immunotherapy, which have greatly improved patient quality of life and survival. Nevertheless, the response rates are still unsatisfactory for the presence of side effects and development of resistance mechanisms. In this context, tumor microenvironment has emerged as a factor affecting the responsiveness and efficacy of immunotherapy, and the study of its interplay with the immune system has offered new promising clinical strategies. This review provides a brief overview of the currently available immunotherapeutic strategies for melanoma treatment by analyzing both the positive aspects and those that require further improvement. Indeed, a better understanding of the mechanisms involved in the immune evasion of melanoma cells, with particular attention on the role of the tumor microenvironment, could provide the basis for improving current therapies and identifying new predictive biomarkers.

## 1 Introduction

Despite continuous advances in melanoma research, there are active challenges in the field of its therapeutics, largely due to its ability to resist treatments and spread to other organs (Ferlay et al., 2010; Siegel et al., 2020; Siegel et al., 2023). A recent study predicted that newly diagnosed melanoma cases will surge by over 50% by 2040, underscoring the urgent need for novel therapies to counter this trend (Arnold et al., 2022). Notably, the stage of melanoma at diagnosis largely predicts the treatment outcome. In fact, the prognosis for melanoma is highly favorable when the disease is diagnosed at its onset, while a significant mortality rate with a 5-year survival rate of less than 10% is observed in patients with metastases, showing that metastatic diseases are the main causes of deaths associated with melanoma (Bomar et al., 2019). Currently the standard treatment approaches for melanoma include surgery, targeted therapies, and immune checkpoint blockade, while radiation therapy, chemotherapy, and immune cell-based therapies are used for patients with advanced disease or those who are unresponsive to conventional first-line therapies (Heo et al., 2016). The advent of immunotherapy has revolutionized the concept of drug therapy, opening avenues for more effective and personalized treatments (Di Giacomo et al., 2013; Sanlorenzo et al., 2014). Until now, the evolution of immunotherapy has led to the development of oncolytic virus therapy, tumor microenvironment modulators, and immune checkpoint inhibitors (ICIs) (Gurzu et al., 2018; Knight et al., 2023). Among these, the introduction of ICIs in clinical practice has completely modified melanoma patient management and significantly improved their long-term survival (Weiss and Kluger, 2022). Unfortunately, this kind of treatment often offers only transient benefits to the patients and can also produce toxic effects (Weiss and Kluger, 2022; Wolchok et al., 2022). Thus, there is an urgent need to identify predictive biomarkers of the responses and new rational targets for more effective therapies to overcome immune resistance while minimizing the toxic effects. This review provides an updated overview of recent advances in immunotherapy and future perspectives for treatment of metastatic melanoma.

## 2 Melanoma

A melanoma is a highly metastatic tumor promoted by the uncontrolled proliferation of melanocytes (Gurzu et al., 2018); this tumor is histologically classified according to the tumor, node, and metastasis (TNM) system, where the tumor is staged through specific and universal characteristics such as tumor thickness, ulceration, and mitosis in the lesions. The other parameters used for classification of this malignancy are mainly concerned with its ability to involve the lymph node system and distance of metastases from the primary tumor (Balch et al., 2009; Amin et al., 2017). Many studies have shown that the genesis of melanoma is complex and multistage, involving both environmental and genetic factors. It has been demonstrated that even if many benign lesions present alterations of the v-raf murine sarcoma viral oncogene homolog B (BRAF) in the codon V600E, disease progression is bound to the concomitant alterations in other genes involved in several cellular processes (Pollock et al., 2003; Shain et al., 2015). Indeed, benign nevi remain quiescent for several years, and neoplastic transformation only occurs after genetic mutations against target genes, such as telomerase reverse transcriptase (TERT), cyclin-dependent kinase inhibitor 2A (CDKN2A), phosphatase and tensin homolog (PTEN), neurofibromin 1 (NF1), and KIT proto-oncogene receptor tyrosine kinase (KIT). These genetic alterations are responsible for uncontrolled activation of the MAPK and PI3K pathways that are physiologically involved in cell proliferation and survival (Leonardi et al., 2018).

### 2.1 Risk factors

Ultraviolet (UV) radiation exposure contributes to the development of approximately 60%–70% of melanoma through mutagenic processes, release of reactive oxygen species (ROS), and uncontrolled production of growth factors (Whiteman et al., 2001; Garibyan and Fisher, 2010; Sample and He, 2018). UV radiations are divided into two categories that equally promote the genesis of melanoma: UV-A (315–400 nm) that are indirectly involved in DNA damage through ROS production and UV-B (280–315 nm) that directly promote DNA mutagenesis (Sample and He, 2018). Only 10% of melanoma are associated with hereditary mutations in genes, which are classified as high, medium, and low penetrance genes based on their ability to promote cancer (Tsao et al., 2012; Read et al., 2016; Soura et al., 2016). Among the high penetrance genes, about 40% of all patients with familial melanoma exhibit mutation in the CDKN2A gene. In this regard, two independent studies have shown that CDKN2A^−/−^ mice exposed to UV irradiation or that mice harboring mutations of the HRAS and NRAS genes develop cutaneous melanoma rapidly (Chin et al., 1997; Yang et al., 2007; VanBrocklin et al., 2010). Medium penetrance genes are represented by the melanocortin 1 receptor (MC1R), microphthalmia-associated transcription factor (MITF), and solute carrier family 45 member 2 (SLC45A2), which are all involved in skin pigmentation. Pasquali et al. (2015) demonstrated that patients with only a single allelic variation of the MC1R gene showed 40% increased risk of developing melanoma compared to the wild-type control subjects and that the presence of an additional allelic mutation further increased this risk by 28%. Furthermore, multiple allelic changes double the risk of disease onset (Pasquali et al., 2015). The germline variant of MITF (p.E318K) is characterized by replacement of the glutamic acid residue in position 318 with a lysine; this mutation alters the post-translational modification state of MITF by disrupting a conserved SUMOylation site, thus affecting the MITF transcriptional activity (Miller et al., 2005). Many low penetrance genes that are correlated with low probabilities of melanoma onset have been identified by genome-wide association studies (GWASs) (Potrony et al., 2016).

### 2.2 Mutations

Over the recent few decades, both activating and deleterious mutations, including single-nucleotide variants (SNVs, somatic and germline mutations) and copy number variations (CNVs), have been documented extensively as the alterations driving melanoma (Guan et al., 2015). Actual knowledge on these genetic alterations is listed in public databases such as the skin cutaneous melanoma catalog in The Cancer Genome Atlas (TCGA) or cBioPortal for Cancer Genomics (www.cbioportal.org) (Guan et al., 2015; Leonardi et al., 2018). Approximately 60% of melanoma patients present somatic mutations of BRAF, a proto-oncogene that encodes a serine/threonine kinase crucial for the MAPK signaling pathway. *In vitro* studies have shown that BRAF inhibition promotes cell growth reduction and apoptosis induction, while reducing tumor formation *in vivo* (Hingorani et al., 2003; Hoeflich et al., 2006; Hoeflich et al., 2009; Tsao et al., 2012; Leonardi et al., 2018; Savoia et al., 2019). Notably, BRAF-mutated melanoma are associated with shorter survivals in both metastatic and early-stage patients (Long et al., 2011). To date, several drugs targeting BRAF activity have been developed and approved for melanoma treatment (Koelblinger et al., 2018; Roskoski, 2018; Morales et al., 2019). Importantly, BRAF mutation and expression can also modulate the immunological phenotypes of melanoma. Indeed, Tomei et al. (2015) compared BRAF-mutant *versus* wild-type samples and identified two immune‐related phenotypes, among which the poor phenotype characterized by an undifferentiated status and a poor prognosis was associated mostly with BRAF mutations (Tomei et al., 2015). Interestingly, the phosphoinositol-3-kinase (PI3K)/AKT pathway is often associated with resistance to BRAF inhibitors in melanoma (Paraiso et al., 2011; Irvine et al., 2018). Beyond the BRAF mutations, melanoma are frequently linked with PTEN loss; PTEN is involved in the regulation of many cellular processes, such as cell growth, survival, and cell motility, and the concomitant mutations in PTEN and BRAF are associated with reduced overall survival (OS) in 44% of melanoma (Bazzichetto et al., 2019). The missense mutation of neuroblastoma RAS viral oncogene homolog (NRAS) occurs in 15%–20% of all patients and is correlated with a more aggressive melanoma subtype with elevated capacity for metastasis. To date, no specific drugs have been approved for NRAS mutations because several strategies targeting NRAS directly have failed to produce effective therapeutics. Additionally, many clinicians do not routinely perform mutational profiling of NRAS, although its detection could have prognostic implications and facilitate clinical trial enrollment (Randic et al., 2021). Finally, less frequent mutations in other genes, such as the mitogen-activated protein kinase (MEK) involved in constitutive ERK activation and chemoresistance (Nikolaev et al., 2011; Stark et al., 2011), as well as sporadic mutations of KIT, NF1, and TERT have been identified (Beadling et al., 2008; Nagore et al., 2009; Handolias et al., 2010; Whittaker et al., 2013; Nissan et al., 2014).

## 3 Immunotherapy and melanoma

The immune system is an intricate network of organs, cells, and soluble factors involved in the protection of the body. Immunotherapy is a type of cancer treatment that takes advantage of this ability in the development of strategies to counteract cancer growth (Beadling et al., 2008; Handolias et al., 2010; Stark et al., 2011). Studies on tumor evasion mechanisms from the perspective of immune control have allowed the development of several molecular drugs that can “educate” the immune system to recognize and kill tumor cells (Falcone et al., 2020; Herrscher and Robert, 2020). In this context, metastatic melanoma are considered perfect examples of immunogenic tumors owing to the important presence of lymphocytic infiltrates (Sanlorenzo et al., 2014). Before the advent of immunotherapy in 2011, the average life expectancy for metastatic melanoma patients was about 9 months. Today, thanks to the identification of new therapeutic targets and development of new immunotherapy drugs, approximately 20% of melanoma patients survive for up to 10 years after diagnosis (Ascierto et al., 2023a). The immunological approaches for melanoma treatment include ICIs (Lugowska et al., 2018; Simsek et al., 2019; Baltussen et al., 2021); vaccines (Choubey, 2019); biological drugs such as cytokines, stimulating factors, and interferons (Choubey, 2019); as well as adoptive cell therapy (ACT) (Chen and Gao, 2019).

### 3.1 Immune checkpoint inhibitors (ICIs)

ICIs are monoclonal antibodies that were initially developed to bind and inhibit T-lymphocyte antigen 4 (CTLA-4) and programmed cell death protein 1 (PD-1), which are both present on the surfaces of T lymphocytes (Lugowska et al., 2018; Simsek et al., 2019). More recently, inhibitors capable of blocking new immune targets, such as lymphocyte-activation gene 3 (LAG-3), have also been developed (Huard et al., 1995). Table 1 summarizes the most important ICIs involved in the treatment of metastatic melanoma.

**TABLE 1:** Summarizes the most important ICIs involved in metastatic melanoma treatment.

Inhibitor	Target	Class
Ipilimumab (MDX-010)	CLTA-4	Selective human IgG1 monoclonal antibody
Tremelimumab CP-675,206	CLTA-4	Selective human IgG2 monoclonal antibody
Nivolumab (BMS-936558, MDX-1106)	PD-1	Selective human IgG4 monoclonal antibody
Pembrolizumab (MK-3475)	PD-1	Selective humanized IgG4 monoclonal antibody
Pidilizumab (CT-011)	PD-1	Selective humanized IgG1 monoclonal antibody
BMS-936559 (MDX-1105)	PDL-1	Selective human IgG4 monoclonal antibody
Atezolizumab (MPDL3280A)	PDL-1	Selective humanized IgG1 monoclonal antibody
Durvalumab (MEDI4736)	PDL-1	Selective humanized IgG1 monoclonal antibody
Avelumab (MSB0010718C)	PDL-1	Selective humanized IgG1 monoclonal antibody
AMP-224	PD-1	PDL-2 fusion protein
AMP-514	PD-1	PDL-2 fusion protein
Relatlimab (BMS-986016)	LAG-3	Selective human IgG4 monoclonal antibody
Fianlimab (REGN3767)	LAG-3	Selective human IgG4 monoclonal antibody
RO7247669	PD-1/LAG-3	Bispecific antibody

#### 3.1.1 CTLA-4 inhibitors

**FIGURE 1:**
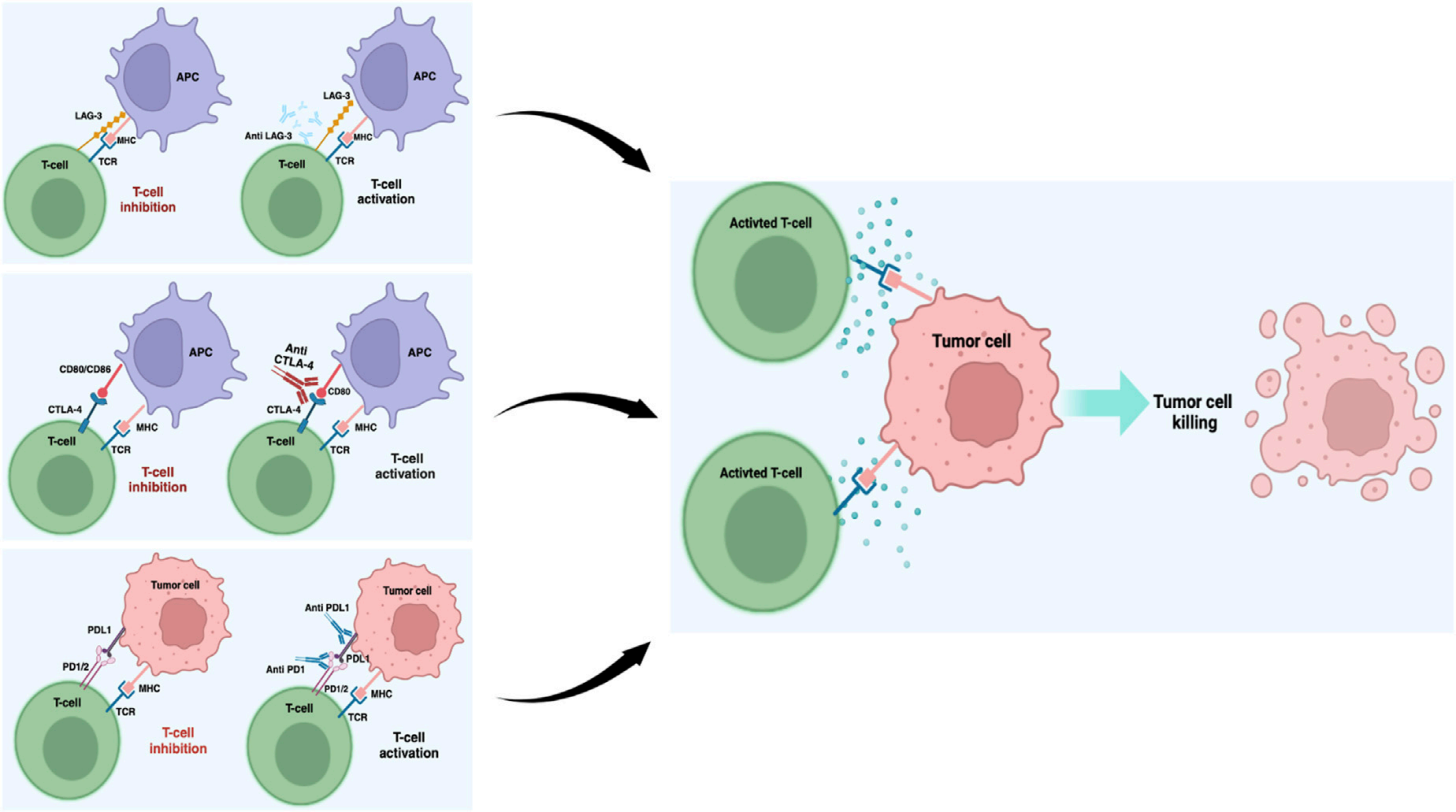
Schematic representation of the most important ICI blockades in melanoma. Under normal conditions, T cell activation is provided by the MHC and TCR signaling pathways, while these pathways are suppressed by the ICIs working together in the tumor microenvironment. ICIs block the interactions between checkpoints and their partner proteins, thus preventing the off signals from being sent, consequently allowing the T cells to kill the cancer cells. This image was created with BioRender (https://biorender.com).

CTLA-4 is a transmembrane receptor belonging to the immunoglobulin superfamily and is present on both CD4^+^ and CD8^+^ T lymphocytes. After binding with the receptors B7-1 (CD-80) or B7-2 (CD86) expressed on the antigen-presenting cells (APCs), CTLA-4 promotes inhibitory signals that regulate T cell activities (Figure 1). Thus, CTLA-4 is an important pharmacological target in the treatment of many neoplastic forms, including metastatic melanoma (Franklin et al., 2017; Khair et al., 2019; Kim and Choi, 2022). The discovery of ipilimumab (MDX-010), an IgG1 monoclonal antibody for CTLA-4, has greatly improved the treatment outcomes of metastatic melanoma, and its use in combination with PD-1 inhibitors has helped achieve a previously unimaginable increase in the OS. The CheckMate 067 trial showed that approximately half of the patients receiving first-line metastatic melanoma treatment as a combination of nivolumab (PD-1 inhibitor) and ipilimumab were alive after 7.5 years. The median OS with the combination treatment was 72.1 months compared to the 36.9 months OS for the PD-1 inhibitor and 19.9 months OS with ipilimumab alone. In addition, the treatment efficacy was maintained over the long term (Hodi et al., 2018; Turajlic et al., 2018; Wolchok et al., 2022). Although research has promoted the development of other CTLA-4 inhibitors, their results have not been as remarkable as those obtained with ipilimumab. For example, no differences were observed in terms of OS between patients treated with the fully human IgG2 monoclonal antibody tremelimumab (CP-675206) and those receiving chemotherapy or other immunotherapy agents (Ribas et al., 2019; Hamid et al., 2023).

#### 3.1.2 PD-1 axis inhibitors

PD-1 is a protein that is physiologically involved in immune system repression and is activated by two ligands: PD-1 ligand (PDL-1) and PD-2 ligand (PDL-2). When tumor cells interact with PD-1 on T cells via PDL-1, the PI3K/AKT pathway is inhibited, leading to cell cycle arrest and T cell activity inhibition (Buchbinder and Desai, 2016) (Figure 1). Melanoma is frequently characterized by high PDL-1 expression, and different antibodies blocking the PD-1/PDL-1 axis, such as nivolumab (BMS-936558, MDX-1106) and pembrolizumab (MK-3475), have been approved for its treatment (Francisco et al., 2010; Hino et al., 2010; Pardoll, 2012; Sunshine and Taube, 2015; Li et al., 2016). These two selective IgG4 monoclonal antibodies, when used alone or in combination with other immunotherapy agents, have shown important results in terms of progression-free survival (PFS) and OS in many clinical studies conducted on metastatic melanoma patients (Robert et al., 2015; Tsai and Daud, 2015; Simeone and Ascierto, 2017; Amaria et al., 2022; Tawbi et al., 2022; Wolchok et al., 2022; Ascierto et al., 2023a; Patel et al., 2023). In particular, the CheckMate 037 clinical trial showed that in metastatic melanoma patients in whom ipilimumab or BRAF inhibitor therapy have failed, nivolumab promoted an objective response rate (ORR) of 31.7% as opposed to 10.7% in patients receiving chemotherapy (Weber et al., 2015). Similarly, in other clinical trials, previously untreated melanoma patients were treated with both nivolumab and ipilimumab alone or in combination; the median PFS and ORR were significantly improved in both the combination and nivolumab-only groups compared to the ipilimumab-only group (Larkin et al., 2015). Recently, an interesting study showed that immunological analysis conducted after a single anti-PD-1 dose can predict patient clinical outcome (Huang et al., 2019); the authors observed rapid immune responses after PD-1 blockade mediated by T cell reactivation and a complete or major pathologic response in 30% of the patients within 3 weeks. These patients had 100% disease-free survival at 24 months, contrary to patients without significant pathologic responses who had poor prognosis with more than 50% recurrence despite therapy at the time of surgery/treatment (Huang et al., 2019). Therefore, a neoadjuvant treatment protocol could allow early identification of patients at high risk of recurrence, thus suggesting transition to a more effective therapy. The advent of pembrolizumab, a monoclonal antibody acting against the PD-1 protein, has become a therapeutic hope for treated patients. In the Keynote-001 trial, approximately 173 melanoma patients resistant to ipilimumab therapy were treated with pembrolizumab at different doses (2 mg/kg every 3 weeks or 10 mg/kg every 3 weeks); these patients showed significant results in terms of survival with ORR of 26% at both doses, with 58% and 63% of patients being alive at the end of 1 year, respectively (Robert et al., 2014). Several studies have also evaluated the activities of other PD-1 axis inhibitors, such as pidilizumab (CT-011), which is a humanized IgG1 monoclonal antibody that has achieved encouraging results regarding inhibited tumor growth and metastasis in preclinical studies in the context of different tumors, including melanoma (Niezgoda et al., 2015). Notably, the inhibition of PD-1 ligands also appears to achieve important results in clinical settings, and several molecules like anti-PDL-1 and PDL-2 have been developed for melanoma treatment. BMS-936559 (MDX-1105) is a fully human IgG4 monoclonal antibody that inhibits binding between PDL-1 and its receptor; similarly, atezolizumab (MPDL3280A), durvalumab (MEDI4736), and avelumab (MSB0010718C) are three humanized IgG1 monoclonal antibodies with high affinities and specificities to PDL-1 that are already used in several clinical trials for metastatic melanoma (Li et al., 2016; Keilholz et al., 2019; Blank et al., 2021; Mao et al., 2022). Finally, in addition to antibody-based treatments, two PDL-2 fusion proteins (AMP-224 and AMP-514) capable of inhibiting PD-1 have been developed (Sunshine and Taube, 2015).

#### 3.1.3 LAG-3 inhibitors

LAG-3 is a CD4 homolog expressed on T lymphocytes that binds the major histocompatibility complex MHC-II, thus inhibiting T cell proliferation and activity (Huard et al., 1995; Demeure et al., 2001; Camisaschi et al., 2014). Several studies have shown the presence of a LAG-3-positive lymphocytic infiltrate in melanoma, laying the basis of a new therapeutic approach aimed at LAG-3/MCH-II binding inhibition (Hemon et al., 2011; Durham et al., 2014). In 2022, the US Food and Drug Administration (FDA) approved relatlimab (BMS-986016) as the first human IgG4 monoclonal antibody anti-LAG-3 for the treatment of several cancers, including melanoma (FDA, 2022). The RELATIVITY-020 study conducted by Ascierto et al. (2023b) on 518 melanoma patients showed that the combination of relatlimab and nivolumab had satisfactory and durable clinical results in patients with metastatic melanoma that were previously treated with PDL-1 inhibitors; indeed, the median PFS here ranged from 2.1 to 3.2 months (95% CIs, 1.9 to 3.5 and 1.9 to 3.6, respectively), and the PFS rates at 6 months ranged from 27.7% to 29.1% (95% CIs, 20.5 to 35.4 and 24.2 to 34.1, respectively) across patient cohorts characterized by different treatment doses (Ascierto et al., 2023b). The RELATIVITY-047 study then analyzed 714 untreated melanoma patients who were divided into two groups equally to receive a combination of relatlimab and nivolumab or nivolumab alone. The study showed positive results in terms of the median PFS for the combination therapy compared to monotherapy with nivolumab (10.2 and 4.6 months, respectively). The study also evaluated the correlations between the PDL-1 and LAG-3 expression levels and treatment responses. Patients with LAG-3 expressions ≥1% presented ORR improvements with the combination than monotherapy with nivolumab (47% and 35%, respectively). On the other hand, LAG-3 expressions <1% resulted in ORRs of 31% and 24%, respectively. Similar ORR results were also obtained by assessing the PDL-1 expression levels: when PDL-1 was ≥1%, the ORR was 53% in the combination case and 45% in monotherapy; when PD-L1 was <1% the ORRs were 36% and 24%, respectively (Tawbi et al., 2022). Despite the positive results, the combination vs nivolumab alone also showed frequent adverse events (81.1% vs 69.9%, respectively). The fully human IgG4 fianlimab (REGN3767), which is another LAG-3 inhibitor, has been utilized in combination with cemiplimab (PD-1 inhibitor) in patients with advanced melanoma. In this trial, the investigators observed ORRs of 63.6% in the PDL-1 naïve patients and 13.3% in patients previously treated with a PDL-1 inhibitor; they showed that the combination was associated with a good safety profile (ClinicalTrials, 2024). Finally, RO7247669 is a new PD-1/LAG-3 bispecific antibody that blocks PD-1 interactions with PDL-1 and PDL-2 as well as the interaction of LAG-3 with MHC-II; although both findings need further confirmations, this agent appears to have encouraging antitumor activity (Jiang et al., 2021).

## 4 Vaccines

**FIGURE 2:**
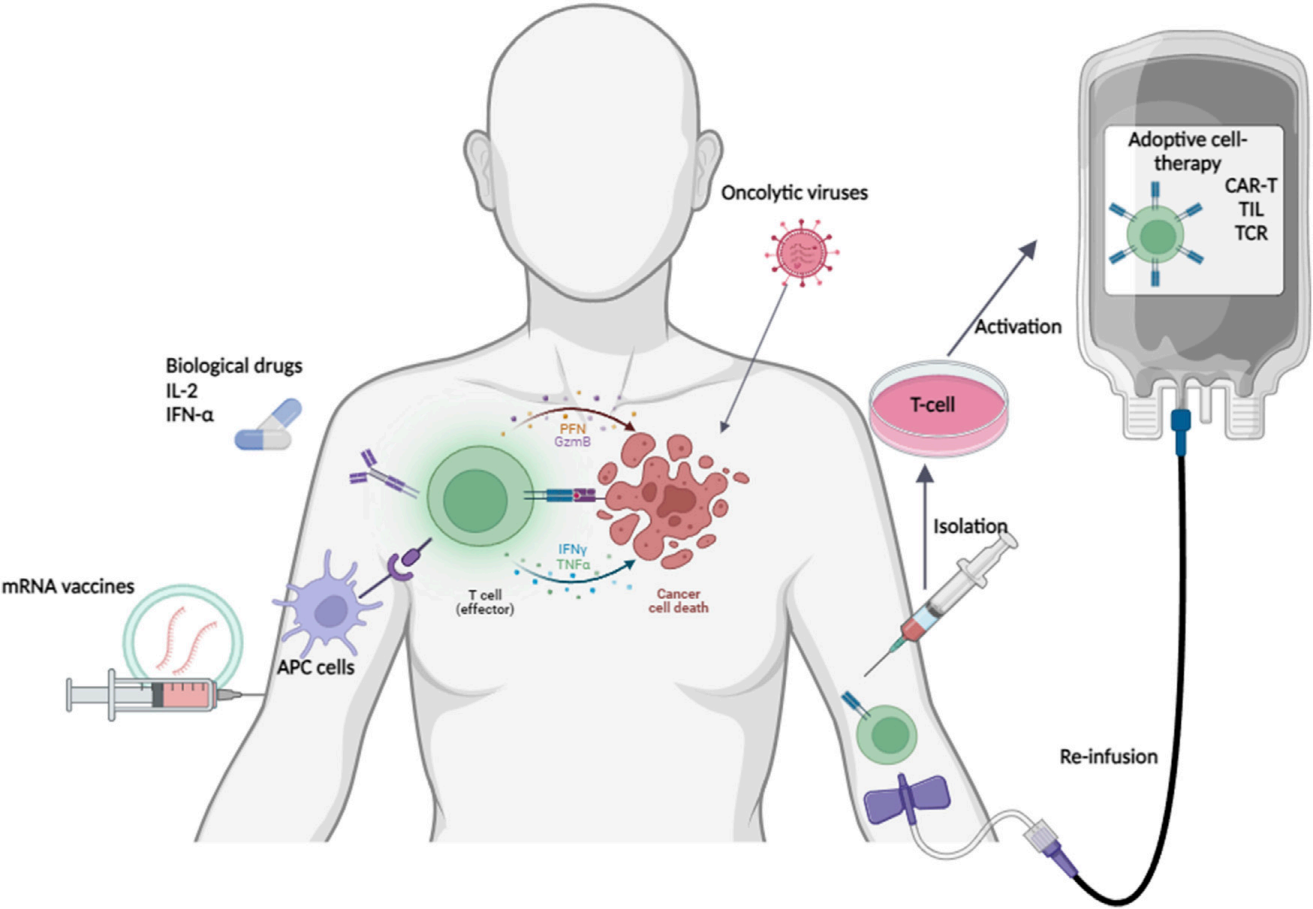
Alternative immunotherapy approaches in melanoma. Although still evolving, alternative immunotherapeutic approaches to ICIs are gaining ground in the treatment of several tumor types, such as metastatic melanoma. This image was created with BioRender (https://biorender.com).

New mRNA vaccines are the latest trend in oncology therapy and exploit the ability of the immune system to recognize and destroy cancer cells (Figure 2). Although this approach is still evolving, alternative immunotherapeutic approaches to ICIs have gained popularity in the treatment of several tumor types, such as metastatic melanoma. The image in this figure was created with BioRender (https://biorender.com).

Vaccines essentially comprise neoantigens, mutated proteins expressed only by tumor cells, tumor-associated antigens present in both normal and tumor cells whose expressions change substantially between them, as well as inflammatory mediators, such as cytokines and chemokines (Liu et al., 2017; Mohan et al., 2018; Xie et al., 2023). Melanoma is the first type of cancer for which these new immunological therapies have been developed and improved, although the results obtained to date are not entirely satisfactory. Glycoprotein 100 (Gp-100) is an important example of a tumor-associated antigen that is highly expressed in melanocytes and melanoma (Wagner et al., 1997). A synthetic peptide formed by a few amino acid residues of its sequence represents the first vaccine formulated for advanced melanoma treatment (Vigneron et al., 2004). *In vivo* experiments have demonstrated satisfactory results in terms of prolonged OS and reduced tumor growth after direct administration of the Gp-100 vaccine (Oberli et al., 2017). In 2011, approximately 185 metastatic melanoma patients were treated with IL-2 alone or in combination with the Gp-100 peptide; the results of this study showed a significant increase in terms of OS for patients receiving combination therapy (Schwartzentruber et al., 2011). De Keersmaecker et al. (2020) instead showed that the use of a next-generation vaccine consisting of Gp-100 and other tumor-associated antigens plus ipilimumab could significantly improve T cell stimulation and the ORR of metastatic melanoma patients (De Keersmaecker et al., 2020). Another vaccine developed for melanoma treatment is Vitespen, a heat shock protein (Gp96)–peptide complex obtained and purified from excised tumors; unfortunately, this did not produce important results in terms of survival in advanced melanoma even while presenting few adverse effects (Testori et al., 2008). The development of new vaccine-based therapeutic approaches is particularly of note as several studies are still open (ClinicalTrials, 2024). One of these is the KEYNOTE-942 study that compares advanced melanoma patients treated with pembrolizumab and a personalized mRNA vaccine, where the patients receive pembrolizumab in monotherapy (Weber et al., 2024). The study recruited 157 patients and divided them into two groups, where 107 received the mRNA vaccine plus pembrolizumab and 50 received pembrolizumab alone, with median follow-up durations of 23 and 24 months, respectively. The recurrence-free survival was noted to be longer with the combination treatment *versus* monotherapy, where the 18-month recurrence-free survival was 79% *versus* 62%, respectively (Weber et al., 2024).

## 5 Other immunotherapy approaches

### 5.1 Biological drugs

The use of immune-stimulating cytokines, such as interleukin-2 (IL-2) and interferon- α (IFN-α), has been approved as adjuvant treatments for melanoma (Figure 2). For many years, it has been noted that high doses of IL-2 appear to activate both T lymphocytes and natural killer (NK) cells, resulting in total response in a small number of the cases. Instead, IFN-α promotes inhibition of tumor proliferation through oncogene repression and activation of the Janus kinase (JAK) signal transducers and activators of transcription (STAT) pathway along with induction of inhibitory chemokine secretion. Unfortunately, the high toxicity associated with both treatments have limited their use to only patients in perfect health conditions (Sanlorenzo et al., 2014; Zhao et al., 2019).

### 5.2 Oncolytic viruses

Oncolytic viruses represent a new and important therapeutic approach that relies on the abilities of the viral particles to replicate only in cancer cells, thus inducing their death (Figure 2). The only approved oncolytic viral treatment of metastatic and advanced melanoma is based on the type I herpes simplex virus and is named Talimogene laherparepvec (TVEC); it has been genetically modified to selectively replicate in cancer cells without infecting normal cells (Liu et al., 2003). TVEC performs two important functions: it acts directly on the tumor cells causing their death and it stimulates the general immune responses of the patient. Several preclinical and clinical studies have shown that the use of TVEC, alone or in combination with other agents (ipilimumab, pembrolizumab), produces encouraging results (Conry et al., 2018).

### 5.3 Adoptive cell therapy

ACT or cellular immunotherapy promotes the therapeutic use of immune cells that are directly isolated from the patients (Figure 2). Upon isolation, these cells can be simply expanded or genetically engineered to enhance their cancer-fighting capabilities (Weber et al., 2020). ACTs are constantly evolving and include different methods: 1) tumor-infiltrating lymphocyte (TIL) therapy; 2) engineered T cell receptor (TCR) therapy; 3) chimeric antigen receptor (CAR) T cell therapy. TIL therapy involves isolating T lymphocytes directly from the patient’s tumor, followed by their clonal expansion *in vitro*. Subsequently, these immune cells are significantly increased in number and can be reinfused into the patient. Although this approach has not yet been approved for clinical practice, it has demonstrated significant responses in patients with advanced melanoma in the Lifileucel study conducted on patients with advanced melanoma that were previously treated by different therapeutic approaches (ICI treatments or BRAF/MEK inhibitors) (Sarnaik et al., 2021). These encouraging results have been confirmed in another study conducted on advanced melanoma cases refractory to anti PD-1 treatments; in this study, 168 patients were equally divided into two groups and treated with TIL or ipilimumab, where patients treated with TIL had a significantly longer PFS of 7.2 months compared to 3.1 months for those who received ipilimumab (Rohaan et al., 2022). In specific cases, T lymphocytes can be engineered to recognize only cancer cells (Zhao and Cao, 2019). In particular, some clinical trials now involve the use of lymphocytes expressing modified TCR, the CAR structured to recognize tumor-specific antigens, in the treatment of patients with metastatic melanoma (Chinnasamy et al., 2010; Chinnasamy et al., 2013; Lu et al., 2017; Yu et al., 2018; Sha et al., 2020). Two studies also evaluated the immune recognition of melanoma-associated antigens by the T cells (MART-1); in both cases, apart from an initial transient tumor regression, no significant results were observed (Abate-Daga et al., 2013; Chodon et al., 2014). Over the past few years, other trials have focused on other melanoma markers, such as the New York esophageal squamous cell carcinoma 1 (NY-ESO-1) and melanoma antigen family A3 (MAGE-A3), obtaining encouraging results in terms of survival. Unfortunately, the small number of patients enrolled or the adverse effects registered subsequently have not allowed us to draw exhaustive conclusions regarding these treatments (Robbins et al., 2015; Lu et al., 2017). Following the important results achieved with hematological diseases, CAR-T therapies have also turned toward the treatment of solid tumors. Several tumor antigens are used as targets for CAR-T cells in the treatment of metastatic melanoma, but preclinical studies have shown encouraging results only for the vascular endothelial growth factor receptor-2 (VEGFR-2). In two independent studies, coadministration of the anti-VEGFR-2 CAR-T cells with exogenous IL-2 or TCR transduced cells significantly increased the tumor-free survival compared to anti-VEGFR-2 CAR-T cells alone in a melanoma murine model (Chinnasamy et al., 2010; Chinnasamy et al., 2013). This *in vivo* finding, unfortunately, did not match the findings of a clinical trial conducted on 24 melanoma patients who received different concentrations of CAR-T cells combined with administration of low or high dose of IL-2. This study was interrupted because of disease progression in substantially all enrolled patients as well as for the occurrence of major adverse effects (Yu et al., 2018). Unfortunately, satisfactory results with CAR-T therapy have not yet been reported for melanoma.

## 6 Melanoma immunotherapy resistance mechanisms

One of the crucial problems of oncology research is to definitively understand the molecular mechanisms underlying resistance to therapies. Although the advent of precision medicine has helped in the development of progressively more targeted and efficient treatments, it has not yet resolved an extremely important question: why are some patients refractory to conventional treatments, or do they develop secondary resistance? Surely, understanding the cellular and molecular mechanisms underlying drug resistance is a demanding challenge owing to the significant intratumor heterogeneities as well as intertumor variations among different patients. Nevertheless, extensive research conducted over the past few years has shed light on some of methods by which melanoma cells can evade immune system surveillance and become resistant to immunotherapy treatments.

### 6.1 Tumor mutational burden (TMB)

TMB represents the total number of somatic mutations per million bases found in a specific tumor. Cutaneous melanoma are known to exhibit higher TMB values than other tumors, mainly due to the C < T transitions caused by UV light, which make them highly immunogenic and therefore ideal for immunotherapy (Sha et al., 2020). Numerous studies have demonstrated significant positive correlations between the TMB of melanoma and immunotherapy responses. For instance, melanoma patients with higher basal levels of mutational burden exhibited improved ORR and PFS when treated with ICIs (Snyder et al., 2014; Ning et al., 2022). Furthermore, a recent study associated TMB with response to adjuvant anti-PD-1 treatment in 165 melanoma patients; in this study, samples were sequenced using a multigene next-generation sequencing (NGS) panel to identify the mutational load before treatment onset, and it was shown that patients with higher TMB values experienced better outcomes and extended relapse-free survival (RFS). The presence of BRAF mutation was also assessed but was identified as an independent predictor of response (Eckardt et al., 2023). Thus, in melanoma, as in other highly immunogenic cancers, the strong correlation between TMB and immune cell infiltration helps clinicians in predicting immunotherapy responses.

### 6.2 Major histocompatibility complex (MHC)

One of the possible mechanisms that promote immune evasion and resistance to treatment involve the reduction or loss of proteins associated with antigen presentation (Lee et al., 2020a; Cornel et al., 2020). A loss of factors associated with MCH-I and II complexes on melanoma cells profoundly reduces responses to ICIs. Indeed, an interesting study on 181 pretreatment melanoma samples showed that a loss of MCH-I is closely related to CTLA-4 inhibitor resistance, while higher levels of MCH-II seem to increase the responsiveness to PD-1 inhibitors (Rodig et al., 2018). This important aspect was also validated in other cancer settings (Alspach et al., 2019).

#### 6.3 PDL-1

The use of PDL-1 expression as a predictive biomarker for the responses of ICIs is still a controversial topic. In fact, PDL-1 expressions vary between the primary tumor and metastases, and even patients with low PDL-1 levels may respond well to therapy (Herbst et al., 2014). Additionally, the parameters used to assess PDL-1 levels have not yet been clearly delineated (Yang et al., 2021). In general, patients with high tumor levels of PDL-1 seem to respond better to PD-1/PDL-1 inhibitors, whereas increased expression of PD-1 on the immune cells negatively regulates treatment responses (Tang et al., 2018).

### 6.4 Circulating biomarkers

#### 6.4.1 Circulating tumor cells (CTCs)

Circulating tumor cells (CTCs) represent a very small percentage of cells in the bloodstream and provide important information on the tumor of origin as well as the metastatic sites (Ricciardi et al., 2023). As a window into the tumor, the analysis of CTCs could be a valid clinical tool for the evaluation of therapeutic approaches toward establishing more personalized medicine. In fact, it has been demonstrated that melanoma patients with PD-L1^+^ CTCs are eight times more likely to respond to pembrolizumab than patients with undetectable PD-L1^+^ CTCs (Khattak et al., 2020). Moreover, the development of a CTC gene signature in the context of melanoma has promoted early assessment of a long-term immunotherapy response (Hong et al., 2018).

#### 6.4.2 Circulating tumor DNA (ctDNA)

All cells, including cancer cells, physiologically release DNA into the bloodstream after apoptotic or necrotic processes. Therefore, the circulating tumor DNA (ctDNA), which are essentially small fragments of genetic material, can be considered good indicators of therapeutic responses as their concentrations in the blood vary over time after treatment (Ricciardi et al., 2023). Many studies have shown significant negative correlations between ctDNA levels and responses to long-term treatments, such as immunotherapy. Indeed, melanoma patients with higher baseline ctDNA levels have presented lower average OS because of poor responses to treatments (Lee et al., 2020b; Marczynski et al., 2020; Marsavela et al., 2020).

#### 6.4.3 Circulating tumor microRNAs (ctmiRNAs)

Although represented at low concentrations, circulating tumor microRNAs (ctmiRNAs) have been evaluated as predictive biomarkers of treatment responses in several cancer settings, including melanoma (Valihrach et al., 2020; Huang et al., 2022). For example, an interesting research has shown that in patients with advanced melanoma, several miRNAs (miR-4649-3p, miR-1234-3p, and miR-615-3p) show upregulation after treatment failure with ICIs (Bustos et al., 2020).

### 6.5 Epigenetic modifications

Epigenetic modifications induce changes in DNA accessibility and chromatin structure, which affect the phenotype without altering the DNA sequence (Handy et al., 2011). Cancer cells frequently experience epigenetic events, leading to changes in the gene activation levels. Deregulation of DNA methylation is considered an epigenetic modification (Yang and Wang, 2021), and it has been amply demonstrated that many tumor phenotypes can be attributed to promoter hypermethylation of tumor-related genes. In melanoma, the most common event is the methylation of CpG islands at the gene promoters, which negatively affect the genes involved in differentiation, replication, tumor suppression, and immune antigen presentation, including Ras-association domain family 1 isoform A (RASSF1A), O6-methylguanine-DNA methyltransferase (MGMT), cyclin-dependent kinase inhibitor 2A (CDKN2A), and PTEN (Esteller et al., 2001; Ehrlich, 2009; de Unamuno Bustos et al., 2018; Aleotti et al., 2021). Several lines of evidence have shown that melanoma cells can downregulate MHC levels by inducing less permissive chromatin states to evade immune surveillance (Garrido et al., 2010). Consistently, HDAC inhibition restores MHC expression in the murine B16 melanoma cells and in human melanoma (Khan et al., 2008; Sarkar et al., 2015). Several studies have also demonstrated that the hypermethylation levels of certain genes could be used to differentiate melanoma patients from healthy individuals and that they can serve as diagnostic markers. Indeed, aberrant DNA methylation is an early event in carcinogenesis and could be revealed by liquid biopsy. Interestingly, the progression of melanoma can lead to changes in the DNA methylation patterns; thus, longitudinal monitoring of DNA methylation in a non-invasive manner through liquid biopsy can provide real-time information on the behaviors and stages of melanoma (Salvianti et al., 2015; Diefenbach et al., 2020).

### 6.6 Immunosuppressive microenvironment

Cancer cells promote an immunosuppressive microenvironment necessary for their survival and escape drug interventions. In this protumoral context, multiple components of the immune system undergo significant changes that alter their anticancer capabilities. For instance, T lymphocytes experience altered differentiation, leading to upregulation of several inhibitory receptors on their surfaces, including PD-1, CTLA-4, LAG-3, immunoglobulin, mucin domain-containing molecule 3 (Tim3), and the immunoreceptor tyrosine-based inhibition motif (ITIM) domain (TIGIT) protein (Tian et al., 2015; Falcone et al., 2020). These dysregulated T cells facilitate tumor immune escape and consequently contribute to immunotherapy resistance (Chauvin et al., 2015; Noyes et al., 2022). Alterations in energy metabolism, particularly lipid metabolism, are fundamental for the development of these exhausted and dysregulated T cells. Owing to their hyperproliferative properties, cancer cells exhibit increased energy demands, and this persistent demand can result in significant modifications to the other components of the tumor microenvironment (TME) over time. For example, elevated cholesterol levels in the TME induce dysregulation in the T cells and lead to increased expressions of immune checkpoint proteins on their surfaces (Ma et al., 2019; Ma and Yi, 2019). T regulatory cells (Tregs), which are a subpopulation of T cells with oncogenic characteristics, play crucial roles in the maintenance of the TME. When present, Tregs induce anticancer immunity (Viehl et al., 2006; Ascierto et al., 2010), and their depletion enhances the immune responses while improving the therapeutic outcomes (Zappasodi et al., 2018). Cancer cells also manipulate cytokine expressions to create more favorable TMEs. Melanoma cells, along with other solid tumors, release many proinflammatory cytokines and soluble factors that increase immunological tolerance within their TMEs. For instance, the production of VEGF or transforming growth factor β (TGF-β) attracts immunosuppressive myeloid-derived suppressor cells (MDSCs) to the tumor sites, thereby promoting treatment resistance (Umansky et al., 2014). Moreover, specific tumor-derived micro-RNAs (miRNAs) have been shown to promote the transformation of macrophages to M2-like cells that interact with T cells to induce an immunosuppressive microenvironment refractory to the anti-PD-1 agents (Umansky et al., 2014).

## 7 Combinational therapeutic approaches

In light of the above discussion, it is clear that immunotherapy, like other treatments, is susceptible to the tumor’s defense mechanisms that can fail over time in most patients. Therefore, in many cases, the adoption of different therapeutic approaches that are able to target multiple tumor vulnerabilities provides better OS. Specifically, in melanoma, the combination of immune and targeted therapies is a promising option. Indeed, several preclinical studies have shown that in a murine model of BRAF-mutated melanoma, treatment with BRAF inhibitors improves the antitumor effects of TCR-engineered ACT, increases the expression of melanoma-associated antigens, and decreases the expression of immunosuppressive cytokines (Koya et al., 2012; Frederick et al., 2013). Conversely, especially in non-response phases, BRAF inhibition promotes immune evasion (Frederick et al., 2013). Several clinical trials have evaluated this combinational treatment strategy in patients with advanced melanoma characterized by BRAF mutations. In all major trials, although characterized by different settings, the triple combination of MAPK inhibitors and ICIs resulted in significant increases in the median PFS values compared to targeted therapy alone (Ferrucci et al., 2020; Dummer et al., 2022; Dummer et al., 2023; ClinicalTrials, 2024). Unfortunately, the appearance of side effects has led to reevaluations of these studies. Although not yet considered in the clinical context, an additional evaluable therapeutic approach for melanoma is the combination of ICIs and poly(ADP-ribose) polymerase (PARP) inhibitors. Indeed, evidence has shown that about 40% of the cutaneous melanoma have homologous recombination DNA damage repair defects that would benefit from the use of PARPi (Peyraud and Italiano, 2020; Chan et al., 2021; Zhou et al., 2023). This combination could have interesting implications in melanoma treatment for several reasons. First, PARP is implicated in not only DNA damage repair but also regulation of immune responses. When DNA damage repair is blocked, the cellular mutational load increases significantly, further exposing the tumors to the protective actions of the immune system. Moreover, there is a compensatory upregulation of PDL-1 on the tumor cells, which results in increased response to ICIs (Chan et al., 2021). Based on the same premise mentioned previously, the combination of immunotherapy and radiotherapy could also have interesting implications. Indeed, one of the effects related to radiotherapy is increased tumor antigen visibility and promoted priming of the T cells. Thus, immunotherapy could be synergized with radiation-induced immune activation to make the microenvironment less favorable for tumor growth (Tagliaferri et al., 2022).

## 8 Personalized treatment approaches

The composition of the TME is emerging as a possible biomarker of response to ICI therapy. Based on the cellular infiltrates, three TME classes have been identified: inflamed (or “hot”) with high levels of intratumoral (IT) and peritumoral (PT) lymphocytes, excluded (or “altered”) with high PT but low IT lymphocytes, and ignored (or “cold”) characterized by the lack of lymphocytes and associated with negative responses to ICI therapy (Sobottka et al., 2021). Indeed, several studies have associated the TME composition with patient responses to ICI treatment. Gide et al. (2019) performed a multifactor analysis to identify genes expressed differentially between patients responding or not responding to the combined anti-CTLA-4 and anti-PD-1 immunotherapy; they found upregulation of the T-cell-related genes and genes associated with NK-cell-mediated cytotoxicity as well as increased T-cell cytotoxicity and cytokine signaling in responders compared to non-responders (Gide et al., 2019). Furthermore, recent evidence suggests that the maintenance of an immunosuppressive TME can also be influenced by the presence of tumor-associated macrophages (TAMs) (Qian and Pollard, 2010). Arlauckas et al. (2017) used time-lapse microscopy in a mouse model of anti-PD-1 responsive cancer and demonstrated that TAMs are able to capture anti-PD-1 mAbs from the T cell surfaces, thus decreasing their efficacy (Arlauckas et al., 2017). In addition, anti-PD-1 mAbs bound to the receptors of the macrophages are able to promote T-cell-mediated cytotoxicity, weakening the effectiveness of the therapy (Zhang et al., 2018). Interestingly, several studies have also hypothesized that the gut microbiome may influence response to immunotherapy by producing short-chain fatty acids (SCFAs) that impact the epigenome of the melanoma cells (Lam et al., 2021). In this context, Luu et al. (2021) showed that the SCFA pentanoate increases the antitumor activity of the CD8^+^ T cells by inhibiting the class I histone deacetylase.

## 9 Toxicity and quality of life

In recent years, there has been an increasing focus on not only improvement of survival outcomes but also health-related quality of life (HRQoL), which can be seen as a set of long-term aspects concerning the social, psychological, emotional, and cognitive statuses of the patient that are impacted by the treatments (Mamoor et al., 2020; Pinto et al., 2022). Indeed, despite the obvious advantages, treatment with ICIs is related to several toxic effects called immune-related adverse effects (irAEs) owing to immune activation and inflammatory responses against the healthy tissue of the host. These toxic effects may affect multiple organs, such as the skin, liver, lungs, and colon, leading to severe declines in their functions and sometimes fatal outcomes (Wang et al., 2018). Moreover, the appearance of side effects is linked to the patient’s state of health. Previous reports have shown that patients with preexisting anti-thyroglobulin antibodies are more sensitive to developing thyroid disorders upon treatment with ICIs (Kimbara et al., 2018). Similarly, a correlation between CTLA-4 gene polymorphism or cytokine activity and development of irAEs after anti-CTLA-4 treatment has been demonstrated. For example, high levels of IL-17 were found in patients who developed colitis following anti-CTLA-4 treatments. The possibility of emerging toxicities due to combinational therapies also needs particular attention. The effects of the combination of ipilimumab and nivolumab on HRQoL have been reported by two research groups from the CheckMate 067 study (Larkin et al., 2019; Schadendorf et al., 2023); these studies found that there was were no clinically meaningful differences in HRQoLs in patients receiving the combination therapy compared to those receiving nivolumab monotherapy.

## 10 Future perspectives and conclusions

The immune system is the first line of defense against anything deemed “foreign” to the human body. Notably, tumors are formed by cells that can be considered “foreign” in many aspects as they acquire peculiar and specific characteristics that are not present in normal cells. With this in mind, the strategy of harnessing and amplifying the capabilities of our immune system to recognize and block tumor proliferation has revolutionized cancer therapy. Metastatic melanoma was the most dismal among solid tumors until a few decades ago, with survival chances of only a few months after diagnosis. Although excisional surgery and conventional chemotherapy were effective strategies for most patients, they did not provide satisfactory results against this highly heterogeneous tumor (Falcone et al., 2020). The advent of targeted therapies has opened new therapeutic avenues, but immunotherapy in particular revolutionized the treatment landscape of melanoma by improving not only the life expectancy but also overall conditions of the patients. However, the potential for toxic effects and resistance to treatments necessitates identification of predictive biomarkers that have till date only allowed creation of individualized therapies specific to each patient’s tumor and immune landscape. Several putative predictive biomarkers have been proposed, but none of these possess enough sensitivity and specificity when used alone, and only combinations of multiple biomarkers have been shown to predict the effectiveness of immunotherapy. This approach should guide clinicians to better stratify patients to achieve control over the disease for a longer period and to overcome the innate and acquired resistances to immunotherapy. Additionally, to determine the optimal therapy regimen, the presence of comorbidities and patient’s quality of life must be considered, as some effective immunotherapies can induce high levels of toxicity. Specifically, patients with comorbidities should be rigorously monitored for possibly increased toxicity. For example, a recent study by Mallardo et al. (2023) showed that cotreatment with nivolumab and relatlimab could have reduced efficacies in patients affected by type 2 diabetes, probably due to the reduced expression of LAG-3 (Mallardo et al., 2023). Another area that remains to be explored concerns the role of the gut microbiome in the interplay between the host and cancer, and the response to immunotherapy thereof. Microbiota are influenced by several factors, and their understanding and manipulation via fecal transplantation or dietary settings could have interesting therapeutic implications. In fact, it has been demonstrated that fecal transplantation can change the gut microbiome, thereby reprogramming the TME and reducing the resistance to anti-PD-1 treatment (Davar et al., 2021; Bolte et al., 2023). To date, one of the popular topics regarding the progression of metastatic melanoma concerns identification of factors that can predict patient responses to ICIs. Recently, omics technologies have provided very powerful tools for discovering novel biomarkers/signatures to predict responses to cancer treatments. For example, the IFN-gamma signature has been identified to predict responses to ICIs in melanoma patients (Grasso et al., 2020; Yan et al., 2021). However, a holistic approach is necessary to achieve this purpose, in which gut microbiome characterization together with genomics, transcriptomics, and immunological insights could provide solid assistance in predicting the outcomes of immunotherapies.
